# Effect of polygenic scores of telomere length alleles on telomere length in newborns and parents

**DOI:** 10.1111/acel.14241

**Published:** 2024-06-28

**Authors:** Yunsung Lee, Astanand Jugessur, Håkon K. Gjessing, Jennifer R. Harris, Ezra Susser, Per Magnus, Abraham Aviv

**Affiliations:** ^1^ Centre for Fertility and Health Norwegian Institute of Public Health Oslo Norway; ^2^ Department of Global Public Health and Primary Care University of Bergen Bergen Norway; ^3^ Mailman School of Public Health Columbia University, and New York State Psychiatric Institute New York New York USA; ^4^ Center of Human Development and Aging, New Jersey Medical School Rutgers University Newark New Jersey USA; ^5^ Department of Pediatrics, New Jersey Medical School Rutgers University Newark New Jersey USA

**Keywords:** genetic nurturing, leukocyte telomere length, newborn, Norwegian Mother, Father and Child Cohort Study, polygenic score, sex, Southern blot, triad design

## Abstract

In adults, polygenic scores (PGSs) of telomere length (TL) alleles explain about 4.5% of the variance in TL, as measured by quantitative polymerase chain reaction (qPCR). Yet, these PGSs strongly infer a causal role of telomeres in aging‐related diseases. To better understand the determinants of TL through the lifespan, it is essential to examine to what extent these PGSs explain TL in newborns. This study investigates the effect of PGSs on TL in both newborns and their parents, with TL measured by Southern blotting and expressed in base‐pairs (bp). Additionally, the study explores the impact of PGSs related to transmitted or non‐transmitted alleles on TL in newborns. For parents and newborns, the PGS effects on TL were 172 bp (*p* =  2.03 × 10^−15^) and 161 bp (*p* =  3.06 × 10^−8^), explaining 6.6% and 5.2% of the TL variance, respectively. The strongest PGS effect was shown for maternally transmitted alleles in newborn girls, amounting to 214 bp (*p* =  3.77 × 10^−6^) and explaining 7.8% of the TL variance. The PGS effect of non‐transmitted alleles was 56 bp (*p* = 0.0593) and explained 0.6% of the TL variance. Our findings highlight the importance of TL genetics in understanding early‐life determinants of TL. They point to the potential utility of PGSs composed of TL alleles in identifying susceptibility to aging‐related diseases from birth and reveal the presence of sexual dimorphism in the effect of TL alleles on TL in newborns. Finally, we attribute the higher TL variance explained by PGSs in our study to TL measurement by Southern blotting.

## Graphical abstract

This study examined the impact of polygenic scores (PGSs) on telomere length (TL) in newborns and their parents, using Southern blotting for measurement. The PGS effect was 172 bp in parents and 161 bp in newborns, explaining 6.6% and 5.2% of TL variance, respectively. Notably, strong PGS effect was shown for maternally transmitted alleles in newborn girls, amounting to 214 bp and explaining 7.8% of the TL variance. Our findings highlight the importance of TL genetics in understanding early‐life determinants of TL.

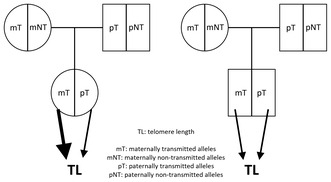

## 1 INTRODUCTION

Telomere length (TL) affects the risk of many diseases and influences longevity in humans (Arbeev et al., 2020; Codd et al., 2021). It is largely heritable (Hjelmborg et al., 2015; Slagboom et al., 1994), shortens with age (Aubert et al., 2012; Factor‐Litvak et al., 2016; Steenstrup et al., 2017), and displays significant inter‐individual variation already at birth (Factor‐Litvak et al., 2016). After birth, telomeres shortening generally follows a fixed tracking pattern, such that individuals born with either short or long telomeres tend to maintain their TL ranking, that is, having shorter or longer TL at a given age, throughout life (Benetos et al., 2013, 2019). Consequently, in middle‐to‐high‐income societies, where most telomere research has been performed, TL ranking is primarily determined at birth. This conclusion has significant implications for human health.

Genome‐wide association studies (GWASs) have identified many TL‐associated alleles in adults. These alleles have been used to construct polygenic scores (PGSs) (Codd et al., 2021). Although these PGSs explain less than 5% of the TL variance in adults, they are valuable in predicting the risk of a host of diseases, including cancer and atherosclerotic cardiovascular disease (CVD) (Codd et al., 2021), two disease categories that greatly influence lifespan in middle‐to‐high‐income societies. Therefore, the development of PGSs based on TL alleles has opened a new phase in telomere research, enabling deeper insights into the biological underpinnings of TL and their impact on human diseases and longevity.

Given that TL ranking in humans is primarily established at birth (Factor‐Litvak et al., 2016), we sought to investigate the extent to which TL alleles, identified in adults and assembled into PGSs, affect TL in newborns. As TL PGSs predict adult disease susceptibility, it is vital to determine the variability in and effect size on LTL they explain in newborns, who typically have long telomeres. Understanding this can help assessing the potential of TL PGSs in predicting disease susceptibility from birth. In this context, we also examined parent‐of‐origin effects, that is, whether TL alleles inherited from mothers may have a different impact on newborn's TL than when the same alleles are inherited from fathers. Moreover, recent research suggests that parental genotypes may affect certain traits in the offspring through the non‐transmitted alleles (Kong et al., 2018; Wang et al., 2021). We thus constructed separate PGSs of transmitted and non‐transmitted parental TL alleles and examined their independent effects on newborn's TL. To achieve these objectives, we leveraged TL measurements by Southern blotting (SB) and SNP‐array genotype data from newborn‐parent triads (henceforth ‘triads’) who participated in the Norwegian Mother, Father, and Child Cohort Study (MoBa). Our approach enables quantifying the influence of TL alleles on both parental and offspring TL, expressed in the number of base pairs (bps) attributed to this effect.

## 2 MATERIALS AND METHODS

### 2.1 Study population

MoBa is a nationwide Norwegian pregnancy cohort that recruited 95,200 pregnant women. It includes 114,500 newborns of these mothers and 75,000 of the newborns' fathers (Magnus et al., 2016). During enrollment between 1999 and 2008, blood samples were collected from the mothers both at the 17th week of gestation and at birth, from the fathers at the 17th week of maternal gestation, and from the newborns (cord‐blood) at birth (Paltiel et al., 2014; Ronningen et al., 2006). For our current analysis, newborn‐parent triads were selected based on the following criteria: (1) nulliparous women who gave birth to a singleton baby after a pregnancy free from complications such as gestational diabetes, hypertension, and preeclampsia, and (2) newborns with no apparent disease or major congenital anomalies (Figure S1—Supplementary File S1).

This study was approved by the Regional Committee for Medical and Health Research Ethics (REK) South‐East A (reference number 2016/2043) in Norway. Data collection by MoBa was conducted in accordance with the Norwegian Data Protection Agency and after approval from REK was secured. Written consents were obtained from the MoBa participants.

### 2.2 TL measurements

Leukocyte TL measurements were performed in duplicate by SB of the terminal restriction fragments (Kimura et al., 2010). In our study, the intraclass correlation coefficient between duplicate runs performed on different gels was 0.97.

### 2.3 Genotyping

In MoBa, 11,490 triads have been genotyped using different genotyping arrays, including the Illumina HumanCoreExome‐12 v.1.1, HumanCoreExome‐24 v.1.0, and Global Screening Array v.1.0 (Helgeland et al., 2022). Variants with call low rates (<98%), poor signal intensity (cluster separation <0.4), low quality scores (10% GenCall score <0.3), and those deviating from Hardy–Weinberg equilibrium (*p* < 1 × 10^−6^) were excluded. Pre‐phasing was done using SHAPEIT v2.790. Imputation was performed by the sanger imputation service using the positional Burrows–Wheeler transform method and the haplotype reference consortium v1.1 as a reference panel. The end product provided by the sanger imputed service was phased and imputed data in variant call format where the first alleles in newborns are transmitted from fathers, and the second alleles are transmitted from mothers. All annotations were based on the genome reference consortium human build 37. Hereafter, this quality‐controlled genotype dataset is referred to as ‘MOBAGENETICS‐V1’. The criteria for sample selection in MOBAGENETICS‐V1 are detailed in the ‘Selection Criteria for MOBAGENETICS‐V1’ of Supplementary File S1.

### 2.4 PGSs of TL alleles

Of the 197 TL‐associated SNPs reported by Codd et al. (2021), we identified 148 independent SNPs in MOBAGENETICS‐V1 (Helgeland et al., 2022). We imputed 26 of the missing SNPs by selecting neighboring SNPs within a one mega‐base pair region that were in high linkage disequilibrium (LD, *R* ^2^ > 0.9) with the missing SNPs. The LD blocks were inferred using reference data of European ancestry from the 1000 Genomes phase 3 (Genomes Project et al., 2015). The remaining 23 SNPs either were absent from the 1000 Genomes phase 3 or lacked neighboring SNPs with an *R* ^2^ > 0.9. In total, we identified 174 LTL associated SNPs but excluded two on the X chromosome. The remaining 172 autosomal SNPs, which showed a high degree of independence in MOBAGENETICS‐V1 (Figure S2—Supplementary File S1), were used to build PGSs for parents and newborns using the estimated effect sizes (“BetaEuro”) provided by Codd et al. (2021). Here, we define PGS as the weighted sum of effect alleles with the abovementioned BetaEuro as weights. For the newborns, we constructed two PGSs, one based on transmitted parental TL alleles, and one based on non‐transmitted parental TL alleles. Each PGS was standardized to have a mean of zero and a variance of one. Detailed information on the 197 TL‐associated SNPs can be found in Supplementary File S2. The column named ‘Type’ indicates whether each SNP existed in MOBAGENETICS‐V1 or was imputed by its neighboring SNP. The columns to the right of the ‘Type’ column contain relevant information about the imputed SNPs. We did not convert the effect alleles to TL‐increasing alleles. This conversion would merely change the scale of the PGS but would not affect the interpretation of the results. The effect size represents the change in TL per one standard deviation increase in the respective PGS.

### 2.5 Estimates of effects of TL alleles on TL

As a first step, we removed sex and age (or gestational age (GA)) effects from TL by regressing TL on sex and age in parents and newborns, separately. To estimate the effect of TL alleles on TL, we fit linear regression of the residuals resulting from the first step, that is, sex and age (or GA)‐adjusted TL, on each PGS with adjustment for the mode of conception (natural versus assisted reproductive technology) and the top 10 principal components. In newborns, we also estimated a potential effect of parental TL alleles by regressing sex and GA‐adjusted newborn TL on their PGSs of transmitted and non‐transmitted parental TL alleles. Genetic relatedness among the 599 newborns was negligible; the largest kinship coefficients detected was 0.19, as determined by the Kinship‐based INference for GWAS (Manichaikul et al., 2010), implying that the newborns were genetically independent (few sibships). To test the differences in effect sizes of the PGS of transmitted versus non‐transmitted alleles on newborn TL, and the differences in effect sizes between boys and girls, we used resampling methods, that is, bootstrapping and permutation tests, and interaction term of PGS and sex, respectively.

## 3 RESULTS

### 3.1 Participants

TL was measured in 1312 triads whose general characteristics are summarized in Table S1—Supplementary File S1. Of these, 643 triads were previously genotyped and phased (Helgeland et al., 2022) (Figure S3—Supplementary File S1). We excluded 29 triads due to Mendelian inconsistencies (Figure S4—Supplementary File S1) and 15 triads of non‐European ancestry (Figure S5—Supplementary File S1). Our study sample thus comprised 599 triads.

### 3.2 TL characteristics

**FIGURE 1:**
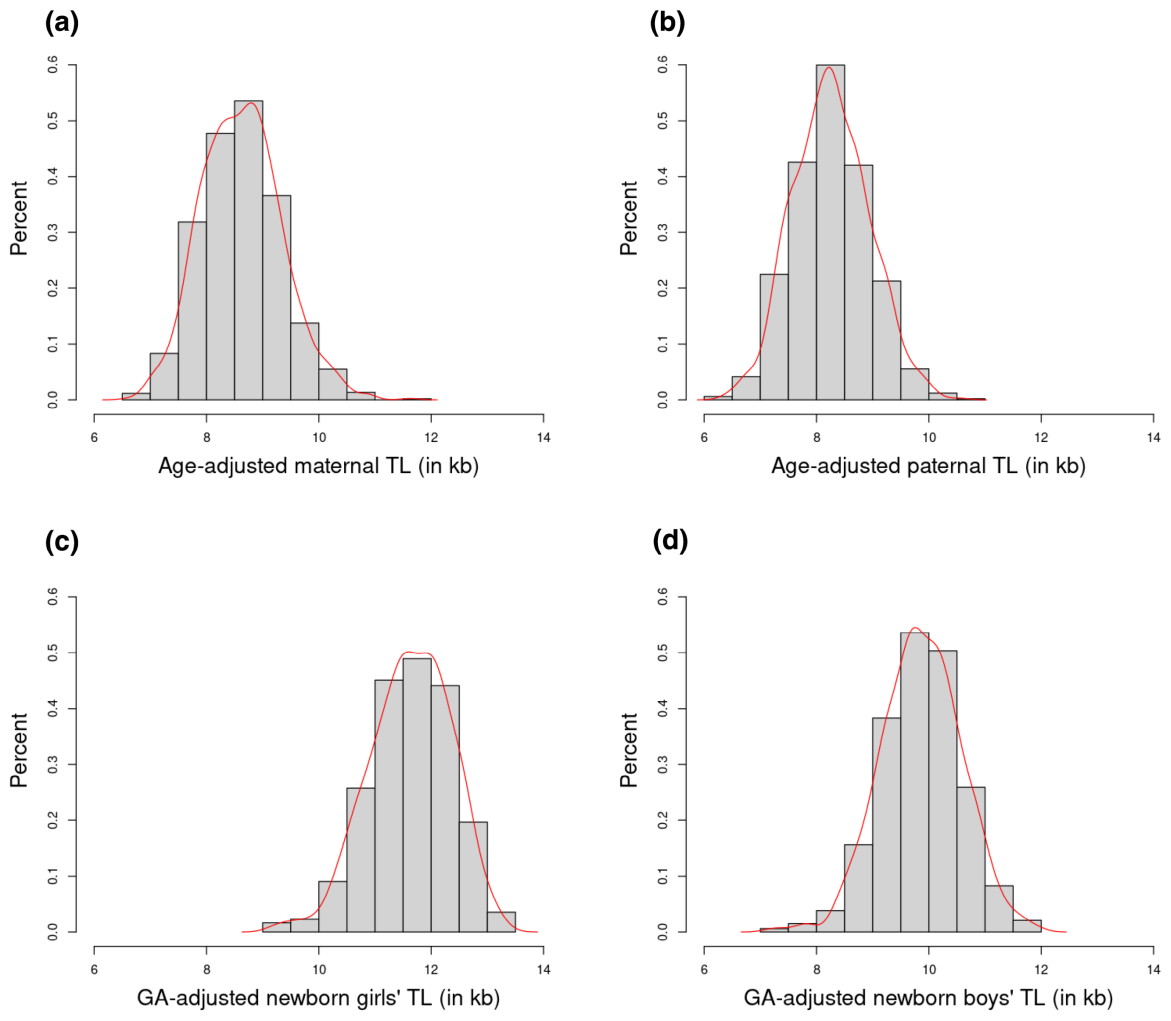
Distributions of age (or GA)‐adjusted TL in parents and newborns. Distributions of age‐adjusted (a) maternal and (b) paternal TL, and GA‐adjusted (c) newborn girls' TL and (d) newborn boys' TL. The mean age‐adjusted maternal TL was 45 bp (*p* = 0.149) longer than the mean age‐adjusted paternal TL. GA‐adjusted newborn girls' TL was 126 bp (*p* = 0.002) longer than GA‐adjusted newborn boys' TL. GA, gestational age; TL, telomere length.

We analyzed data of TL measurement in 1312 triads. Variation in TL was similar for parents and newborns (Figure 1; Table S1—Supplemental File S1). In parents, sex‐adjusted TL was shorter in older individuals (−22 bp/year, *p* = 2.5 × 10^−12^), with mothers having slightly longer age‐adjusted TL than fathers, although the difference was not statistically significant. The shortening of telomeres with age in mothers was −25 bp/year (*p* = 4.3 × 10^−7^), while that in fathers was −16 bp/year (*p* = 2.5 × 10^−5^). The difference between mothers and fathers was not significant (*p* = 0.121). The TL of 918 mothers whose blood samples were taken at the 17th week of gestation was not different from that of 142 mothers whose blood samples were taken at birth (7810 bp vs. 7827 bp, respectively; *p* = 0.790). Age‐adjusted maternal and paternal TL was moderately correlated (Pearson's correlation (*r*) = 0.293, *p* = 4.4 × 10^−19^). In newborns, those with a longer GA had shorter TL (−37 bp/week, *p* = 0.020), and girls had longer GA‐adjusted TL than boys (difference = 126 bp, *p* = 0.002). Newborns with older fathers had longer TL than those with younger ones (11 bp for each additional year in fathers older than 18 years, *p* = 0.025).

Significant correlations were observed between parental TL and newborn TL (for mothers, *r* = 0.376, *p* = 1.3 × 10^−36^; for fathers, *r* = 0.360, *p* = 3 × 10^−32^). However, the two correlations were not significantly different from each other (*p* = 0.599). On average, parental TL was 1428 bp shorter than that of the newborns (*p* < 2 × 10^−16^).

### 3.3 Effect of TL alleles on TL in parents and newborns

Table 1 shows the estimated effect sizes of the PGSs on TL and the proportions of variance explained in parents. The overall PGS effect in parents amounted to 172 bp, explaining 6.6% of the TL variance, meaning that the mean parental TL was increased by 172 bp per SD increase in parental PGS. When the analyses were stratified by parental sex, the PGS effect was 207 bp in mothers, explaining 8.9% of the TL variance, while the PGS effect was 135 bp in fathers, explaining 4.5% of the TL variance. However, the difference in the PGS effects between mothers and fathers was not statistically significant (*p* = 0.097).

**TABLE 1:** Effect of PGS for TL on TL in parents.

	PGS	
	Effect size^c^	Variance explained^d^
Sex and age‐adjusted parental TL (*n* = 931)^a^	172 bp	6.6%
	(130, 214)	
	(*p* = 2.03 × 10^−15^)	
Age‐adjusted maternal TL (*n* = 476)^b^	207 bp	8.9%
	(147, 267)	
	(*p* = 5.46 × 10^−11^)	
Age‐adjusted paternal TL (*n* = 455)^b^	135 bp	4.5%
	(77, 193)	
	(*p* = 5.94 × 10^−6^)	

Abbreviations: PGS, polygenic scores; TL, telomere length.

^a^ Sex and age‐adjusted TL was regressed on the PGS with adjustments for mode of conception (natural versus assisted reproductive technology) and the top 10 principal components.

^b^ Age‐adjusted TL was regressed on the PGS with adjustment for the same covariates as above.

^c^ The numbers in parentheses refer to 95% confidence intervals and *p*‐values.

^d^ Partial *R* ^2^ for the respective PGS term.

Table 2 summarizes the effects of the PGS on the sex‐ and GA‐adjusted TL in newborns. This effect size was 161 bp, explaining 5.2% of the TL variance, which is similar to that observed in the parents. However, the effect of the PGS of transmitted maternal TL alleles was stronger (151 bp, variance explained 4.5%) than that of transmitted paternal TL alleles (79 bp, variance explained 1.3%). This difference was especially evident in newborn girls, with the PGS effect of 214 bp, variance explained 7.8%, for the transmitted maternal TL alleles compared to 72 bp for the transmitted paternal TL alleles (the difference being 142 bp, *p* = 0.019).

**TABLE 2:** Effect of PGS of transmitted TL alleles on newborn TL.

		PGS of transmitted alleles	Maternal	Paternal
Sex and GA‐adjusted TL (boys and girls, *n* = 593)^a^	Effect size^c^	161 bp	151 bp	79 bp
		(105, 218)	(95, 208)	(22, 136)
		(*p* = 3.06 × 10^−8^)	(*p* = 2.06 × 10^−7^)	(*p* = 0.007)
	Variance explained^d^	5.2%	4.5%	1.3%
GA‐adjusted TL (girls, *n* = 275)^b^	Effect size^c^	187 bp	214 bp	72 bp
		(102, 271)	(125, 303)	(−12, 157)
		(*p* = 2.18 × 10^−5^)	(*p* = 3.77 × 10^−6^)	(*p* = 0.095)
	Variance explained^d^	6.7%	7.8%	1.1%
GA‐adjusted TL (boys, *n* = 318)^b^	Effect size^c^	140 bp	104 bp	92 bp
		(63, 216)	(30, 178)	(13, 171)
		(*p* = 4.04 × 10^−4^)	(*p* = 0.006)	(*p* = 0.024)
	Variance explained^d^	4.0%	2.4%	1.7%

Abbreviations: PGS, polygenic scores; TL, telomere length.

^a^ Sex and GA‐adjusted newborn's TL was regressed on respective PGS with adjustment for mode of conception (natural versus assisted reproductive technology) and the top 10 principal components.

^b^ GA‐adjusted newborn's TL was regressed on respective PGS with adjustment for the same covariates as above.

^c^ The numbers in parentheses refer to 95% confidence intervals and p‐values.

^d^ Partial *R* ^2^ for the respective PGS term.

The overall effect of PGSs of non‐transmitted parental TL alleles on newborns was not significant, although non‐transmitted maternal TL alleles appeared to exert a stronger effect than non‐transmitted paternal TL alleles (Table S2).

We conducted several sensitivity analyses. First, we included two TL‐associated SNPs on the X chromosome in the PGSs and estimated their direct genetic effects on TL in newborns (Tables S3 and S4—Supplementary File S1). The results were almost identical to those obtained using the PGSs without the two X‐chromosome SNPs. Second, we estimated the effect of PGS on TL in mothers by excluding 63 maternal samples collected at birth (most maternal samples were collected at the 17th week of gestation). The effect size increased by 22 bp, and the variance explained increased by 1.9%p. Third, when estimating the effect size of PGS on TL in newborns, we included genotype batch as an additional adjusting variable in the linear regression model, but the results did not change appreciably. Fourth, instead of the two‐stage model (described in the Materials and Methods section), we checked if a one‐stage model, that is, regression of newborn LTL on PGS, GA, newborn sex, the mode of conception, and the top 10 principal components, shows different results. We found that the results did not change.

## 4 DISCUSSION

PGSs of TL alleles and Mendelian randomization analysis in adults (Codd et al., 2021) suggest a causal relationship between TL and multiple TL‐related traits and diseases, such as hematological malignancies, solid cancers, and atherosclerotic CVD (Codd et al., 2021; Telomeres Mendelian Randomization et al., 2017). The inter‐individual variation in TL is principally determined at birth (Benetos et al., 2013, 2019; Factor‐Litvak et al., 2016). Therefore, the findings from the current study are crucial for understanding the role of TL alleles, from birth onwards, in age‐related diseases and longevity in contemporary humans (Arbeev et al., 2020; Codd et al., 2021).

Selection bias, an important consideration in any study, is unlikely to have had any marked influence on our results for two reasons. First, the overall MoBa sample is far more representative of the general population than other studies used for genomic analyses. For example, UK Biobank is based on 5.5% who agreed to participate (Stamatakis et al., 2021), while MoBa is based on 43.5% participation in a well‐defined national population (Nilsen et al., 2009). Moreover, the Medical Birth Registry of Norway and the in‐depth data collection in MoBa enabled us to examine selection bias in MoBa in some detail (Nilsen et al., 2009). Second, the triads in MOBAGENETICS‐V1 were similar to the overall MoBa participants, except for the samples included in TED (see the Materials and Methods section). Although TED oversampled attention deficit hyperactivity disorder (ADHD) case‐children, it constitutes 5.5% of the total. There is no evidence supporting a relationship between newborn TL and childhood ADHD. Hence, our findings could be generalized to the Norwegian population and likely to populations in middle‐to‐high‐income societies but not to other populations, for instance, sub‐Saharan Africans (McQuillan et al., 2024).

The TL characteristics of the triads analyzed in our study are consistent with those observed in a previous study which specifically investigated TL measured by SB in triads comprising pregnant nulliparous women with singleton births and healthy newborns (Factor‐Litvak et al., 2016). Similarities include a mean TL in newborns of around 9500 bp in the prior study and around 9200 bp in our study, along with a TL standard deviation of roughly 700 bp for both newborns and parents. Other similarities included girls having longer TL than boys, and newborns with older fathers having longer TL. However, there were some differences between the two studies. In the previous study (Factor‐Litvak et al., 2016), maternal TL was longer than paternal TL, and the maternal‐newborn TL correlation was much stronger than the paternal‐newborn TL correlation. We observed similar trends in our study, but the difference in the correlations was not statistically significant. In addition, the inverse relationship between newborn TL and GA has also been reported previously (Vasu et al., 2017). We also confirmed the previous findings of the strong correlation between maternal and paternal TL, although the underlying reasons, including shared environment, remain uncertain (Broer et al., 2013). Taken together, our findings on TL in newborns and parents are consistent with those from previous investigations, highlighting the crucial point that the person‐to‐person variation in TL is predominantly established at birth. This conclusion underscores the importance of understanding the extent to which PGSs of TL alleles contribute to TL in newborns.

Our study is small compared to a recent UK Biobank investigation which evaluated the PGS of TL alleles in around 460,000 adults (Codd et al., 2021). The UK Biobank study used quantitative polymerase chain reaction (qPCR) to measure TL as the ratio (T/S) of the telomeric PCR product (T) to that of a single‐copy gene (S). It found that a PGS of TL alleles explained approximately 4.5% of the T/S variance in adults. In contrast, our study, which employed the highly precise SB method to measure TL in 599 triads, showed that PGS of TL alleles explained 6.6% of the TL variance in adults and 5.2% in their newborns. The higher precision of TL measurement in our study might explain the differences observed between the two studies. More importantly, SB data enabled expressing the effect of TL alleles on TL in bp, underscoring the relatively small effects of PGSs of known TL alleles on TL. Despite this small effect, these PGSs have been helpful in predicting risks for TL‐associated human diseases, presumably because they capture genetic predispositions to such conditions. Furthermore, the similar effects of TL alleles on both newborns and their parents imply that environmental influences in middle‐to‐high‐income societies on TL after birth may be minimal (Aviv, 2022; Bountziouka et al., 2022; Pepper et al., 2018).

Interestingly, TL alleles transmitted from mothers had a notably stronger effect on TL in newborns compared to those from fathers. This effect was particularly pronounced in newborn girls. While the underlying reasons behind these findings are yet to be fully understood, it is worth mentioning that past studies have shown a higher correlation between maternal and offspring TL than that between paternal and offspring TL (Broer et al., 2013; Factor‐Litvak et al., 2016). Although we observed a similar pattern in our study, the difference was not statistically significant.

Parental genotypes can potentially affect traits of offspring through the effects of non‐transmitted alleles, a concept referred to as “genetic nurturing” (Kong et al., 2018; Wang et al., 2021). However, our relatively small study of around 600 newborns yielded findings on the potential effect of non‐transmitted TL alleles on newborn TL that are, at best, borderline significant.

We acknowledge additional limitations in our study. First, the triads were exclusively of European ancestry. Second, given our focus on newborns and their young parents, we did not examine data linking our findings to TL‐related health outcomes that typically manifest later in life. Nonetheless, the longitudinal design of MoBa will allow future evaluations of how our findings relate to health outcomes (Ronningen et al., 2006).

In conclusion, our study demonstrates that PGSs of TL alleles in newborns and their parents account for a greater variance in TL than previously reported. In contrast with prior studies, we now quantify this effect in bp and reaffirm the notion that TL in the general population is primarily established at birth. These findings suggest that PGSs of TL alleles may be equally effective in predicting risks of future TL‐associated diseases in adults and newborns. Our study also highlights the greater impact of maternally transmitted TL alleles on TL in newborns, particularly among newborn girls. Finally, further investigations with larger sample sizes are warranted to examine whether non‐transmitted TL alleles may influence newborn TL.

## AUTHOR CONTRIBUTIONS

AA and PM designed the study. YL conducted statistical analyses. AA and YL drafted the manuscript. AJ, HKG, JRH, ES, PM, and AV contributed to the writing of the manuscript and statistical interpretation. AJ, HKG, JRH, PM, and AA acquired funding and administrated the study. All the authors interpreted the results and contributed to manuscript preparation.

## CONFLICT OF INTEREST STATEMENT

The authors declare no conflicts of interest.

## Supporting information


Data S1.

Data S2.

## Data Availability

Access to the genetic datasets can be obtained by applying to the Norwegian Institute of Public Health (NIPH; http://www.fhi.no/en/). Restrictions apply regarding the availability of these data, as they are not publicly available and were used under specific approvals for the current study. Access can be granted only after approval by REK under the provision that the applications are consistent with the given consent. An application form can be found on the NIPH website at https://www.fhi.no/en/studies/moba/. Specific questions regarding access to data in this study can also be directed to Dr. Per Magnus (per.magnus@fhi.no).
