# Supplementary material for: Effect of polygenic scores of telomere length alleles on telomere length in newborns and parents
Source: Aging Cell. 2024 Jun 28;23(9):e14241. doi: 10.1111/acel.14241 (PMC11488311; doi:10.1111/acel.14241)
Supplement: Supplementary file 1 — Data S1. [file ACEL-23-e14241-s001.docx]

Supplementary File 1

**Selection criteria for MOBAGENETICS-V1**

MOBAGENETICS-V1 used the following selection criteria. First, HARVEST excluded those children who were 1) stillborn, 2) deceased, 3) twins, 4) unregistered in Medical Birth Registry of Norway (MBRN), 5) whose anthropometric measurements at birth in MBRN were missing, 6) whose mothers did not answer the first MoBa questionnaire at gestational week 17, or 7) whose parental DNA samples are missing. After applying these exclusion criteria, HARVEST randomly selected mother-father-child triads. Second, ROTTERDAM 1, ROTTERDAM 2, and NORMENT1 adhered to HARVEST’s criteria but ensured their selected triads did not overlap. Third, TED focused on the children whose birth records were available in MBRN, who were liveborn and still alive according to MBRN, and whose DNA samples were available. Then, it selected attention-deficit hyperactivity disorder (ADHD) case-children and their parents and control-children and their parents. Further details on the selection criteria can be found at https://github.com/folkehelseinstituttet/mobagen/wiki/Projects-that-have-contributed-to-MoBa-Genetics. The number of genotyped individuals in each batch is as follows: HARVEST (n=33,538, 33.8%), ROTTERDAM1 (n=17,949, 18.1%), ROTTERDAM2 (n=9,041, 9.1%), NORMENT1 (n=33,321, 33.6%), and TED (n=5,410, 5.5%).


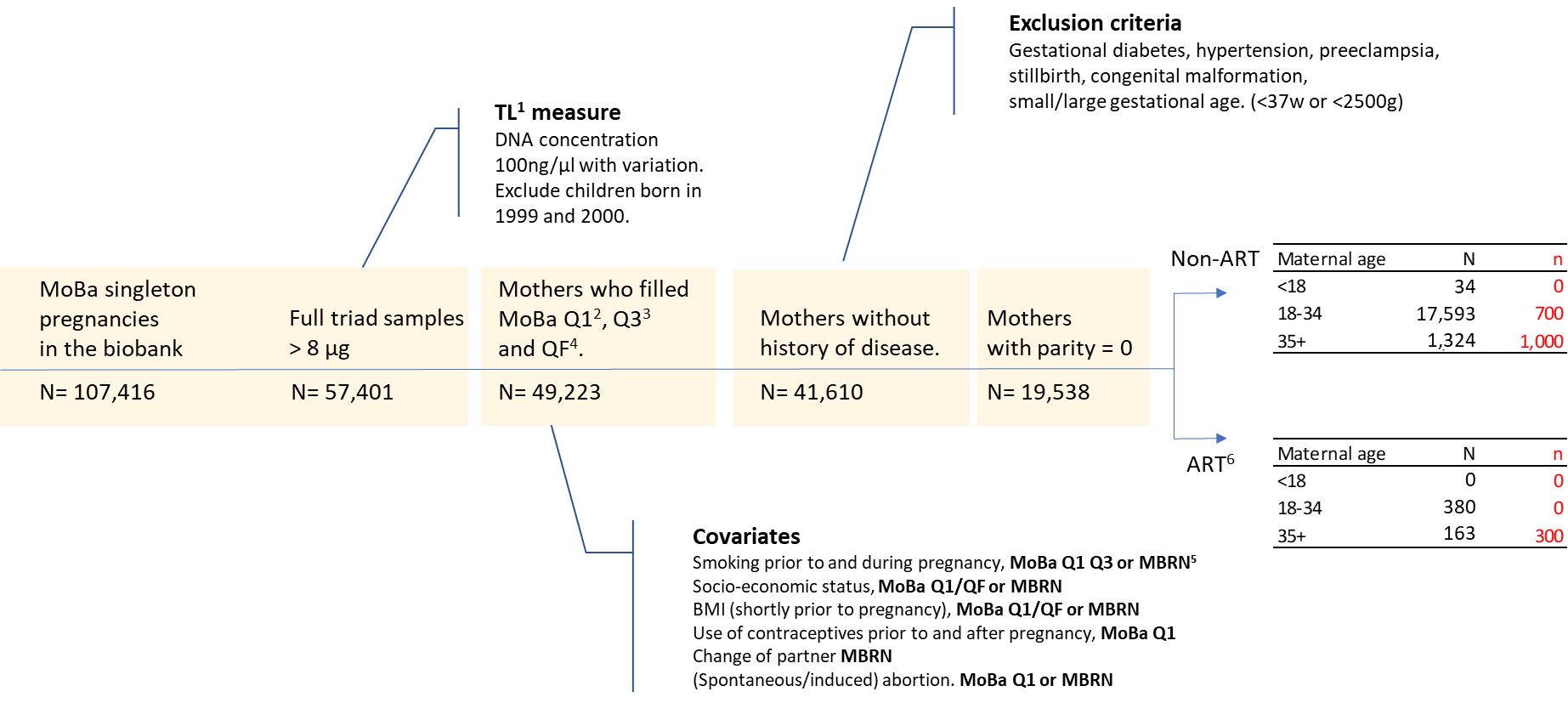


## Figure S1. Sample selection for TL measurements.

The column “N” and “n” of the table displayed on the right side show the number of triads who retained after the exclusion criteria were applied and the number of triads that we aimed to sample (the actual number is slightly different from what was presented in Figure S3). ^1^ Telomere length. ^2^ MoBa Questionnaire collected from mothers at the 17^th^ gestational week. ^3^ MoBa Questionnaire collected from mothers at the 30^th^ gestational week. ^4^ MoBa Questionnaire collected from fathers at the 17^th^ gestational week. ^5^ Medical Birth Registry of Norway. ^6^ Assisted Reproductive Technology.


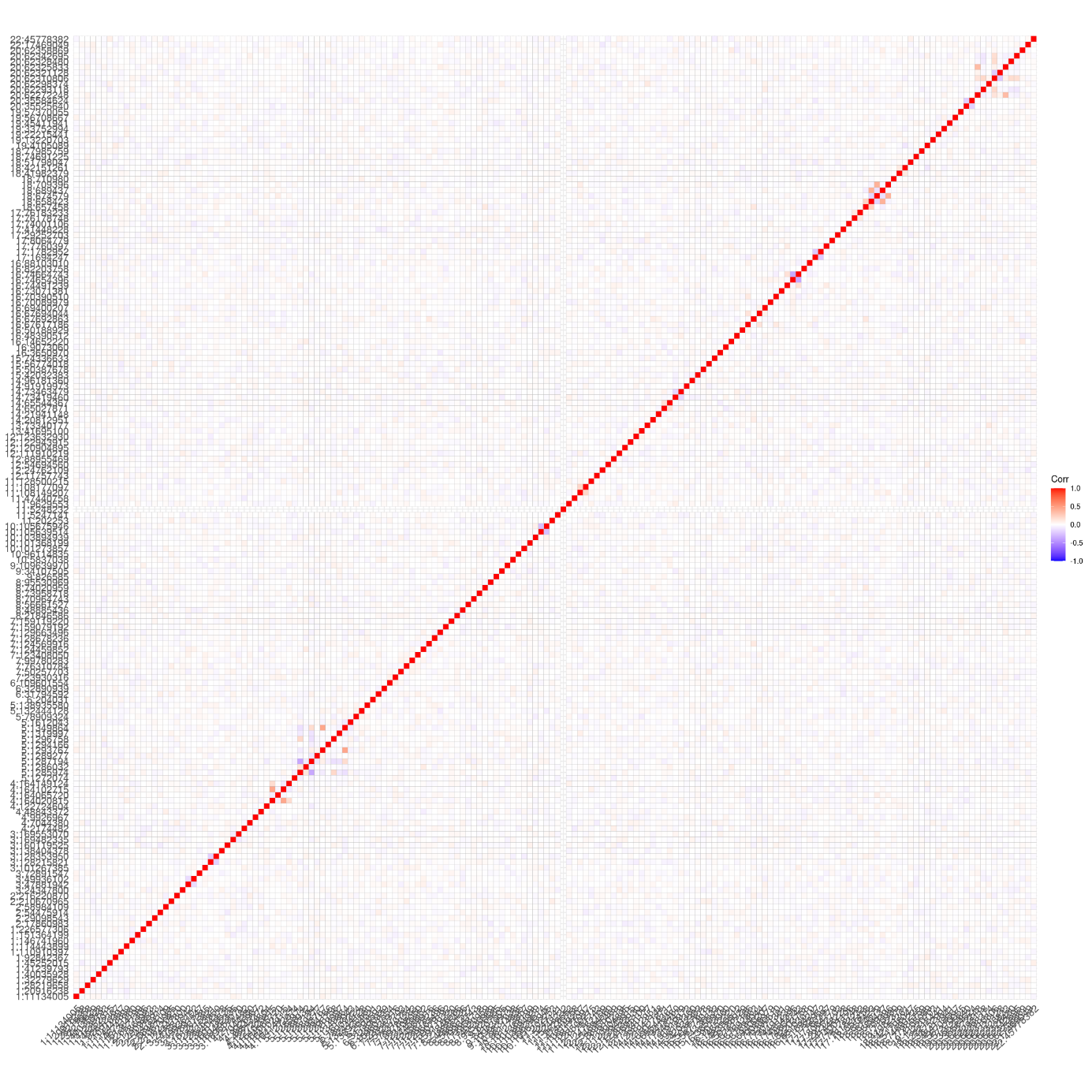


## Figure S2. Correlations of the 172 selected loci for constituting PGS for TL


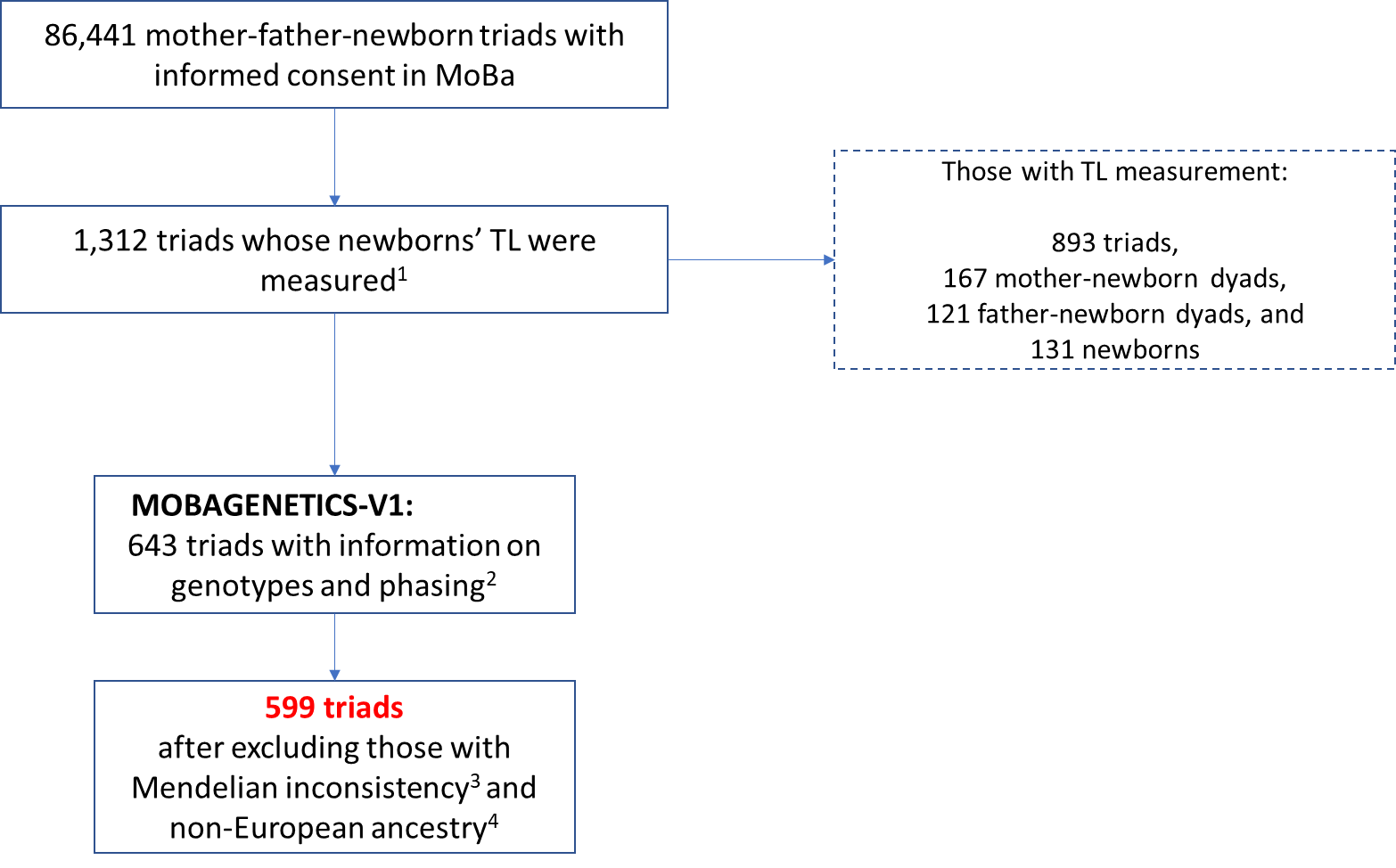


## Figure S3. Flow chart for selected study participants

^1^ Southern blots of terminal restriction fragments proposed by Kimura et al. (Kimura et al., 2010). ^2^ Quality controlled using the MOBAGENETICS-V1 pipeline developed by Helgeland et al. (Helgeland et al., 2022). ^3^ Newborn’s alleles did not exist in parental alleles. ^4^ Estimated using the principal components analysis on participants to MOBAGENETICS-V1 and the HapMap project v3.

## Figure S4. Overview of the 29 triads with Mendelian inconsistency.

For each of 643 triads and each of the 172 SNPs predictive of TL, we verified whether the alleles transmitted to the newborn were present in the parents’ alleles. If not, we marked “x” for the specific triad and SNP. 12 triads showed Mendelian inconsistency across more than one SNP, while the remainders showed Mendelian inconsistency at only one SNP.


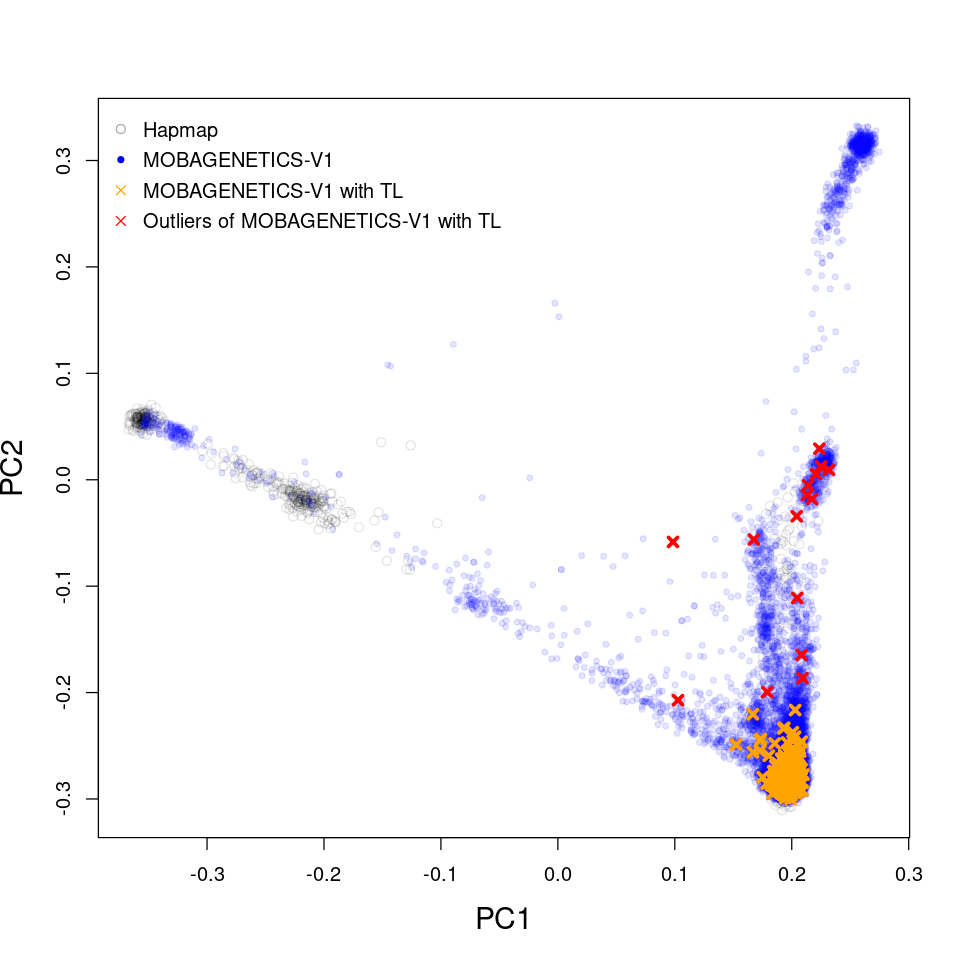


## Figure S5. Principal component analysis on the newborns of MOBAGENETICS-V1 and participants of HapMap project v3

The gray dots refer to the study participants to HapMap project v3, and the blue dots are those to MOBAGENETICS-V1. The crosses (in orange and red) are the MoBa newborns with TL measurement. The orange and red dots represent those of European and non-European ancestry, respectively.

## Table S1. Characteristics of study sample with information on TL and genotypes

|  | N | Mean | SD | Miminum | Median | Maximum |
| --- | --- | --- | --- | --- | --- | --- |
| **Parents** |  |  |  |  |  |  |
| Parental LTL (in kb) | 2,074 | 7.77 | 0.70 | 5.71 | 7.74 | 10.93 |
| Maternal LTL (in kb) | 1,060 | 7.81 | 0.72 | 5.71 | 7.79 | 10.93 |
| Paternal LTL (in kb) | 1,014 | 7.72 | 0.68 | 5.82 | 7.71 | 10.05 |
| Maternal age (in years) | 1,312 | 32.0 | 4.3 | 19.1 | 32.7 | 44.2 |
| Paternal age (in years) | 1,312 | 34.1 | 5.6 | 17.5 | 33.7 | 67.7 |
|  |  |  |  |  |  |  |
| **Newborns** |  |  |  |  |  |  |
| Newborn LTL (in kb) | 1,312 | 9.19 | 0.73 | 6.47 | 9.21 | 11.19 |
| Girls' LTL (in kb) | 625 | 9.26 | 0.74 | 6.78 | 9.31 | 10.99 |
| Boys' LTL (in kb) | 687 | 9.13 | 0.72 | 6.47 | 9.15 | 11.19 |
| Birthweight (in grams) | 1,312 | 3574.2 | 440.6 | 2510.0 | 3549.0 | 5360.0 |
| Gestational age (in weeks) | 1,300 | 40.4 | 1.3 | 36.4 | 40.4 | 43.4 |

## Table S2. Effect of non-transmitted parental TL alleles on newborn TL

|  |  | **PGS of  non-transmitted  alleles** |  |  |
| --- | --- | --- | --- | --- |
|  |  |  | **Maternal** | **Paternal** |
| **Sex and GA- adjusted TL (boys and girls, n=593)^1^** | **Effect size^3^** | 56 bp | 58 bp | 22 bp |
|  |  | (-2, 113) | (0, 115) | (-36, 79) |
|  |  | (p=0.0593) | (p=0.0506) | (p=0.4627) |
|  | **Variance explained^4^** | 0.6% | 0.7% | 0.1% |
| **GA- adjusted TL (girls, n=275)^2^** | **Effect size^3^** | 31 bp | 55 bp | -7 bp |
|  |  | (-56, 118) | (-36, 146) | (-95, 82) |
|  |  | (p=0.4902) | (p=0.238) | (p=0.8858) |
|  | **Variance explained^4^** | 0.2% | 0.5% | 0.0% |
| **GA- adjusted TL (boys, n=318)^2^** | **Effect size^3^** | 82 bp | 64 bp | 45 bp |
|  |  | (3, 162) | (-12, 140) | (-33, 123) |
|  |  | (p=0.044) | (p=0.1019) | (p=0.2582) |
|  | **Variance explained^4^** | 1.3% | 0.9% | 0.4% |

^1^ Sex and GA-adjusted newborn’s TL was regressed on respective PGS with adjustment for mode of conception (natural versus assisted reproductive technology) and the top 10 principal components. ^2^ GA-adjusted newborn’s TL was regressed on respective PGS with adjustment for the same covariates as above. ^3^ The numbers in parentheses refer to 95% confidence intervals and p-values. ^4^ Partial R2 for the respective PGS term.

## Table S3. Effect of transmitted parental TL alleles on both autosomal and X chromosomes on the newborn TL

|  |  | **PGS of  transmitted  alleles** |  |  |
| --- | --- | --- | --- | --- |
|  |  |  | **Maternal** | **Paternal** |
| **Sex and GA- adjusted TL (boys and girls, n=593)^1^** | **Effect size^3^** | 163 bp | 153 bp | 80 bp |
|  |  | (107, 219) | (97, 210) | (23, 137) |
|  |  | (p=2.07E-08) | (p=1.45E-07) | (p=0.006) |
|  | **Variance explained^4^** | 5.3% | 4.7% | 1.3% |
| **GA- adjusted TL (girls, n=275)^2^** | **Effect size^3^** | 186 bp | 215 bp | 70 bp |
|  |  | (101, 271) | (126, 305) | (-15, 155) |
|  |  | (p=2.56E-05) | (p=3.72E-06) | (p=0.108) |
|  | **Variance explained^4^** | 6.6% | 7.9% | 1.0% |
| **GA- adjusted TL (boys, n=318)^2^** | **Effect size^3^** | 142 bp | 107 bp | 92 bp |
|  |  | (66, 219) | (33, 181) | (13, 172) |
|  |  | (p=3.11E-04) | (p=0.005) | (p=0.024) |
|  | **Variance explained^4^** | 4.2% | 2.6% | 1.7% |

^1^ Sex and GA-adjusted newborn’s TL was regressed on respective PGS with adjustment for mode of conception (natural versus assisted reproductive technology) and the top 10 principal components. ^2^ GA-adjusted newborn’s TL was regressed on respective PGS with adjustment for the same covariates as above. ^3^ The numbers in parentheses refer to 95% confidence intervals and p-values. ^4^ Partial R2 for the respective PGS term.

## Table S4. Effect non-transmitted parental TL alleles on both autosomal and X chromosomes on the newborn TL

|  |  | **PGS of  non-transmitted  alleles** |  |  |
| --- | --- | --- | --- | --- |
|  |  |  | **Maternal** | **Paternal** |
| **Sex and GA- adjusted TL (boys and girls, n=593)^1^** | **Effect size^3^** | 54 bp | 57 bp | 20 bp |
|  |  | (-4, 112) | (-1, 115) | (-38, 78) |
|  |  | (p=0.068) | (p=0.053) | (p=0.496) |
|  | **Variance**  **explained^4^** | 0.6% | 0.6% | 0.1% |
| **GA- adjusted TL (girls, n=275)^2^** | **Effect size^3^** | 32 bp | 56 bp | -7 bp |
|  |  | (-56, 120) | (-35, 146) | (-97, 84) |
|  |  | (p=0.477) | (p=0.231) | (p=0.886) |
|  | **Variance**  **explained^4^** | 0.2% | 0.5% | 0.0% |
| **GA- adjusted TL (boys, n=318)^2^** | **Effect size^3^** | 83 bp | 62 bp | 49 bp |
|  |  | (3, 164) | (-14, 138) | (-30, 128) |
|  |  | (p=0.043) | (p=0.113) | (p=0.227) |
|  | **Variance**  **explained^4^** | 1.3% | 0.8% | 0.5% |

^1^ Sex and GA-adjusted newborn’s TL was regressed on respective PGS with adjustment for mode of conception (natural versus assisted reproductive technology) and the top 10 principal components. ^2^ GA-adjusted newborn’s TL was regressed on respective PGS with adjustment for the same covariates as above. ^3^ The numbers in parentheses refer to 95% confidence intervals and p-values. ^4^ Partial R2 for the respective PGS term.

## REFERENCES

Helgeland, O., Vaudel, M., Sole-Navais, P., Flatley, C., Juodakis, J., Bacelis, J., . . . Johansson, S. (2022). Characterization of the genetic architecture of infant and early childhood body mass index. *Nat Metab, 4*(3), 344-358. doi:10.1038/s42255-022-00549-1

Kimura, M., Stone, R. C., Hunt, S. C., Skurnick, J., Lu, X., Cao, X., . . . Aviv, A. (2010). Measurement of telomere length by the Southern blot analysis of terminal restriction fragment lengths. *Nat Protoc, 5*(9), 1596-1607. doi:10.1038/nprot.2010.124
